# Structural Brain Network Abnormalities in Parkinson’s Disease With Freezing of Gait

**DOI:** 10.3389/fnagi.2022.944925

**Published:** 2022-07-08

**Authors:** Chaoyang Jin, Lei Yang, Shouliang Qi, Yueyang Teng, Chen Li, Yudong Yao, Xiuhang Ruan, Xinhua Wei

**Affiliations:** ^1^College of Medicine and Biological Information Engineering, Northeastern University, Shenyang, China; ^2^Key Laboratory of Intelligent Computing in Medical Image, Ministry of Education, Northeastern University, Shenyang, China; ^3^Department of Electrical and Computer Engineering, Stevens Institute of Technology, Hoboken, NJ, United States; ^4^Department of Radiology, School of Medicine, Guangzhou First People’s Hospital, South China University of Technology, Guangzhou, China

**Keywords:** Parkinson’s disease, freezing of gait, diffusion tensor imaging, graph theory analysis, network-based statistic

## Abstract

**Objective:**

Diffusion tensor imaging (DTI) studies have investigated white matter (WM) integrity abnormalities in Parkinson’s disease (PD). However, little is known about the topological changes in the brain network. This study aims to reveal these changes by comparing PD without freezing of gait (FOG) (PD FOG–), PD with FOG (PD FOG+), and healthy control (HC).

**Methods:**

21 PD FOG+, 34 PD FOG-, and 23 HC were recruited, and DTI images were acquired. The graph theoretical analysis and network-based statistical method were used to calculate the topological parameters and assess connections.

**Results:**

PD FOG+ showed a decreased normalized clustering coefficient, small-worldness, clustering coefficient, and increased local network efficiency compared with HCs. PD FOG+ showed decreased centrality, degree centrality, and nodal efficiency in the striatum, frontal gyrus, and supplementary motor area (SMA). PD FOG+ showed decreased connections in the frontal gyrus, cingulate gyrus, and caudate nucleus (CAU). The between centrality of the left SMA and left CAU was negatively correlated with FOG questionnaire scores.

**Conclusion:**

This study demonstrates that PD FOG+ exhibits disruption of global and local topological organization in structural brain networks, and the disrupted topological organization can be potential biomarkers in PD FOG+. These new findings may provide increasing insight into the pathophysiological mechanism of PD FOG+.

## Introduction

Freezing of gait (FOG) is common in the middle and late stages of Parkinson’s disease (PD) (Pardoel et al., 2019). It is a short-lived and intermittent symptom of starting difficulty, causing a great physical and psychological burden on PD patients (Gao et al., 2020). The clinical manifestation of FOG is a short-term block of movement before or during walking. PD with FOG (PD FOG+) patients often feel “sucked on the floor and difficult to lift their feet” (Gonçalves and Pereira, 2013). This situation usually lasts for several seconds, occasionally as long as tens of seconds (Giladi and Hausdorff, 2006). At present, there is no good treatment method for FOG, and traditional medical treatment and electrical stimulation have difficulty achieving similar effects to other symptoms of PD (Giladi, 2008).

Although FOG has devastating consequences for the lives of PD patients, its underlying pathophysiological mechanism is still unclear, and it is called a “mysterious phenomenon” (Nutt et al., 2011; Okuma, 2014). Previous studies have shown that the dysfunction of multiple brain areas, such as the basal ganglia, motor area, frontal and parietal cortex, and brainstem, are important aspects of FOG (Okuma, 2006; Pieruccini-Faria et al., 2015; Dagan et al., 2017). Some studies have reported that due to dysfunction of the cerebral cortex and basal ganglia, the secretion of dopamine in the striatum is reduced, which reduces the activity of the midbrain motor area and leads to FOG (Bartels et al., 2006; Takezawa et al., 2010; Yanagisawa, 2018). In addition, several studies have found that FOG is related to the interaction of the brain’s cognitive network and motor network (Shine et al., 2013; Dagan et al., 2018).

Diffusion tensor imaging (DTI) is a non-invasive magnetic resonance imaging (MRI) method that can show the integrity of nerve fiber bundles and functional area connections in living bodies and can quantitatively assess the degree of white matter damage (Lope-Piedrafita, 2018; Shaikh et al., 2018). Using DTI technology, a series of studies have been conducted to determine whether there is an abnormality in the white matter structure of PD FOG+ patients. Compared with PD FOG-, PD FOG+ patients have shown WM abnormalities in the corpus callosum, cortical white matter tracts of the cingulate gyrus, pedunculopontine nucleus, and supplement motor area (Youn et al., 2012; Iseki et al., 2015; Jin et al., 2021b). Conversely, in terms of tract projection from the supplementary motor area to subcortical areas and whole-brain analysis, no differences were found between PD FOG+ and PD FOG- patients (Fling et al., 2014; Canu et al., 2015). Most studies thus far have focused on identifying white matter lesions in specific regions or fiber tracts, lacking the more complex and in-depth distributed patterns that may exist in DTI data.

Graph theory is a robust mathematical framework for dealing with complex networks, which enables us to quantitatively analyze the topological properties of the brain network and the efficiency of processing information (Bullmore and Sporns, 2009; Sporns, 2018). The brain network constructed by graph theory has some special topological properties, such as small-worldness properties, network efficiency, nodal centrality, and connectivity strength (Sporns et al., 2004; van den Heuvel and Sporns, 2013). The small-worldness properties support segregated, distributed, specialized, and integrated information processing and support high dynamic complexity at minimizing wiring costs, which is very economical (Bassett and Bullmore, 2006). Network efficiency is closely related to the ability of network information transmission. When a complex network has a high parallel information transmission capacity, its network efficiency is often relatively high (Zhang et al., 2018; Chen et al., 2019). Node centrality is a powerful indicator used to evaluate the relative importance of a node in the entire brain network and represents the information integration ability of a single brain region in the entire brain network (Li et al., 2018; Jin et al., 2021a). Changes in topological properties can expose patients to brain network dysfunction or recombination, which may have an impact on the separation and integration of information (Zhang et al., 2017). The exploration of PD FOG+ patients’ structural brain networks will deepen our understanding of the pathophysiological mechanism and promote the implementation of effective treatments.

The graph theory method can be used to explore the changes in the topological properties of the brain network of patients with FOG. Several recent studies have shown that compared with healthy controls (HCs), the topological properties of the brain network of PD patients have undergone overall disruption (Baggio et al., 2015; Kim et al., 2017; Ma et al., 2017). In addition, changes in topological attributes such as global efficiency, local efficiency, and nodal centrality have been related to PD motion subtypes, showing that levodopa is committed to normalizing the damaged network topological organization (Berman et al., 2016; Nambu and Chiken, 2020). However, these studies mainly focused on functional brain networks, and only comparisons between PD FOG- patients and HCs were performed. There are few studies on the structural brain network among PD FOG+, PD FOG- and HC.

In this study, we aimed to explore the abnormality of the topological properties of the structural brain network among the PD FOG+, PD FOG- and HC groups. For this goal, we used DTI images and graph theory to examine the alterations in the entire structural brain network. We hypothesized that PD FOG+ patients would show disrupted structural brain network organization compared to PD FOG- patients and HCs.

## Materials and Methods

### Participants

**FIGURE 1:**
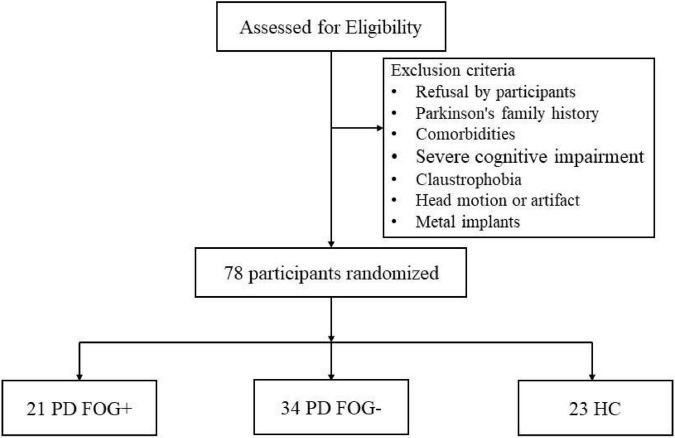
Schematic representation of the inclusion and exclusion criteria for this study. HC, health control; PD FOG- Parkinson’s disease without freezing of gait; PD FOG+, Parkinson’s disease with freezing of gait.

This study included 55 right-handed PD patients. All patients met the diagnostic criteria of the United Kingdom Parkinson’s Disease Society Brain Bank, and the exclusion criteria were as follows (Figure 1): (1) atypical Parkinson’s disease with multiple system atrophy, corticobasal ganglia degeneration, progressive supranuclear palsy, excessive resting tremor, comorbidities, such as Alzheimer’s disease, depression, and anxiety disorders; (2) history of cerebrovascular disease, brain injury, or other neurodegenerative diseases; (3) diseases that seriously affect gaits, such as visual impairment, orthopedic disease, musculoskeletal disease or stroke; (4) severe cognitive impairment or dementia (MMSE score < 24); and (5) serious contraindications to MRI, such as metal implants, claustrophobia, internal devices, etc. A patient who met the following two conditions was considered a PD FOG+ patient: (1) the third item in the FOGQ is greater than one point; (2) more than two experienced neurologists have performed a series of exercise tests (walk, turn around, go through the narrow doorway), and determined that the patient has FOG. Patients who did not meet any of the above conditions were classified as not exhibiting FOG (FOG-). After stopping anti-Parkinson’s disease drugs for at least 12 h to reduce the pharmacological effects on nerve activity, clinical tests and MRI scans were performed after stopping the drugs in the morning.

Twenty-three right-handed HCs were recruited from the community through poster advertisements, including 14 females and 9 males. The following criteria were used to exclude HCs: (1) cognitive impairment (MMSE score is lower than 24); (2) systemic, mental or neurological diseases (such as anxiety, depression, Alzheimer’s, etc.); (3) focal or diffuse brain injury, including lacunar and extensive cerebrovascular diseases; and (4) serious contraindications to MRI, such as metal implants, claustrophobia, internal devices, etc. Finally, 78 participants were included in this study, including 21 PD FOG+ patients, 34 PD FOG- patients, and 23 healthy controls matched by age, sex, and education level. This study was approved by the Ethics Committee of Guangzhou First People’s Hospital and was conducted in adherence with the 1964 Declaration of Helsinki and its subsequent amendments or similar ethical standards. The approved ethics clearance number is K-2018-141-03. All subjects signed an informed consent form before participating in this study.

### Clinical Assessment

All PD patients were assessed for motor and cognitive abilities, especially general cognitive and executive functions. The Unified Parkinson’s Disease Rating Scale (UPDRS-III) and Hoehn and Yahr scale (H&Y) were used to assess the motor disability and severity of PD (Diedrichsen et al., 2011). The Timed Up and Go (TUG) test was used to evaluate mobility, balance, walking ability, and fall risk (Kleiner et al., 2018). The severity of FOG was assessed by employing the FOGQ. The Montreal Cognitive Assessment (MoCA) and MMSE were used to evaluate the intellectual status and cognitive function. The Frontal Assessment Battery (FAB) test was used to assess frontal lobe function (Datta et al., 2019). Subjects also completed the Hamilton Anxiety Rating Scale (HARS) and Hamilton Depression Rating Scale (HDRS) to assess depression and anxiety levels.

### Image Acquisition

MRI data of all subjects are acquired with a 3.0 Tesla MRI system (Siemens Medical Solutions, Germany) equipped with an 8-channel phased-array head coil. When performing MRI scans, all subjects wore earplugs and tight foam head cushions to reduce the effects of scanning noise and head movement. They were told to keep their heads fixed, close their eyes, and not think about anything. Three-dimensional T1-weighted images were acquired using a 3D magnetization-prepared rapid gradient-echo (MP-RAGE) sequence with the following parameters: repetition time (TR) = 1,900 ms, echo time (TE) = 102 ms, flip angle (FA) = 9°, thickness = 1.0 mm, slices = 160, field of view (FOV) = 250 × 250 mm^2^, matrix = 256 × 256, and voxel size = 1.0 × 1.0 × 1.0 mm^3^. Then, the DTI images were acquired using an echo planar imaging (EPI) sequence with the following parameters: TR = 8,700 ms, TE = 102 ms, FOV = 230 × 230 mm^2^, voxel size = 2.5 × 2.5 × 2.5 mm^3^, matrix = 92 × 92, thickness = 2.5 mm, and slice gap = 0 mm. Diffusion gradients are applied in 99 non-collinear directions with a b factor of 2,000 s/mm^2^ after an acquisition with *b* = 0 s/mm^2^ for reference.

### Data Preprocessing

The FMRIB’s Diffusion Toolbox (FDT) and FMRIB’s Software Library (FSL) were used to preprocess DTI data for each subject. Two experienced radiologists performed a visual inspection of the DTI data and T1 data to avoid obvious artifacts caused by subject movement or instrument malfunction. The specific steps of preprocessing DTI data were shown as follows: (1) converting images in DICOM to NIFTI format; (2) b0 image extracting; (3) brain extraction; (4) Correction for eddy currents and head motion by registering the DTI images to the b0 image; and (5) calculation of fractional anisotropy (FA) by using linear least square fitting method.

### Network Construction

The “Pipeline for Analyzing braiN Diffusion imAges (PANDA)” software installed on the Linux system and MATLAB (Cui et al., 2013) was used for network construction. For any effective network organization, nodes and edges are the basic building blocks of graph theory models, so their accurate definition is also very important (Butts, 2009). The automated anatomical labeling template (AAL) divides the entire brain into 90 regions, and then these segmented brain regions will be used as nodes of the brain network so that the constructed brain network has 90 nodes.

The following specific steps are used to define the nodes and edges of the network. (1) Definition of network nodes: In short, register 3D T1-weighted images of each subject to the b0 image in DTI space (non-diffusion weighted gradient direction), and then the transformed T1 images were further non-linearly registered onto the MNI-ICBM152 template. Then, inverse transformations were used to warp the automatic anatomical marker template from the MNI space to the DTI space. In this way, the structural brain network with 90 nodes (45 in each cerebral hemisphere) was acquired. To ensure that the transformation is correct, we check the ROI of each subject in the diffusion space. (2) Network edge definition: The fiber assignment based on the continuous tracking (FACT) algorithm was used for fiber tracking (connecting each pair of brain regions in the diffusion space) and whole-brain fiber construction. When the fractional anisotropy (FA) is less than 0.2 or the tracking turning angular between two connections is greater than 45°, the fiber bundle tracking is stopped. We chose a threshold for fiber bundles to reduce the impact of false connections. If there are at least three fibers (*T* = 3), the two regions are considered structurally connected (network edge).

### Network Analysis

All structural network properties (including global and regional properties) were analyzed by using MATLAB-based GRETNA, a graph theoretical network analysis toolbox (Wang et al., 2015), and visualized by the BrainNet Viewer toolbox (Xia et al., 2013).

First, we analyzed the global properties of the structural brain network. These global properties include small-worldness properties (γ, λ, and δ), clustering coefficient (C_p_), characteristic path length (L_p_), global network efficiency (E_glob_), and local network efficiency (E_loc_). In the small worldness properties, γ, λ, and δ refer to normalized C_p_, normalized L_p_, and small-worldness, respectively. Compared with regular networks, the small-worldness networks have shorter L_p_ and higher C_p_. C_p_ represents the average clustering coefficient of all nodes in the structural network, which is an important indicator to measure the degree of network interconnection. L_p_ is the average shortest path length between any two nodes in the brain network. E_*gloc*_ is considered an important indicator to measure the efficiency of parallel information transmission in the network. E_loc_ measures the communication efficiency among the nodes. When a network possesses these characteristics (γ > > 1, λ≈1, and δ > 1), it is considered to have small-worldness.

**FIGURE 2:**
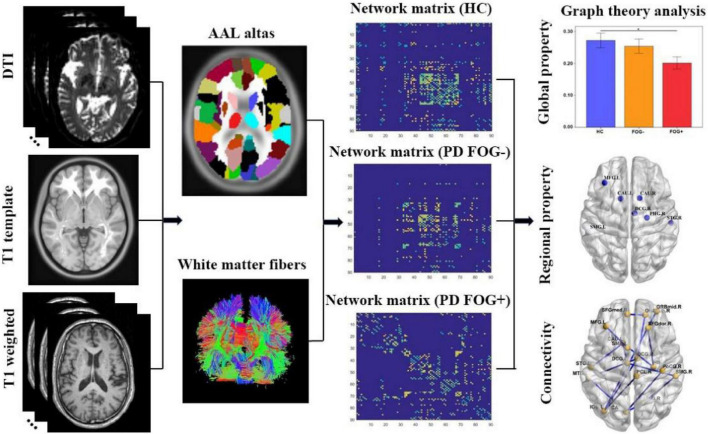
The study procedure of graph theory analysis of structural brain network (DTI, diffusion tensor imaging; AAL, automated anatomical labeling; HC, health control; PD FOG- Parkinson’s disease without freezing of gait; PD FOG+, Parkinson’s disease with freezing of gait).

Second, we investigated the regional properties of the structural brain network among the PD FOG+, PD FOG-, and HC groups. These regional properties include degree centrality (DC), between centrality (BC), node efficiency (NE), node local efficiency (NLE), and nodal shortest path length (N_p_). DC indicates the importance of nodes or brain regions in the whole brain network, while BC indicates the ability of nodes to influence the entire network. Moreover, NE illustrates the efficiency of parallel information transfer for a given node in the brain network, and NLE investigates the transmission capacity of local information in the brain network. N_p_ of a given node quantifies the mean distance or routing efficiency between this node and all the other nodes in the network. The specific processing steps of structural brain network construction are shown in Figure 2.

### Statistical Analysis

The differences in demographic and clinical characteristics between PD FOG+, PD FOG- and HC patients were analyzed through the statistical package IBM SPSS statistics software (SPSS)^1^ for Window version 24.0. One-way analysis of variance (ANOVA) and *post hoc* tests were used to compare variables between three groups. The Mann–Whitney *U*-test was used to compare variables only between two groups. A paired two-sample *t*-test was used to compare the global and local attributes of the structured brain network, taking age, sex, and disease duration into account as covariates. *p* < 0.05 indicated a significant difference. The network-based statistics (NBS) method was used to explore edgewise analyses. In the NBS analysis, non-parametric permutation tests with 10,000 iterations were used to detect significant intergroup differences in structural connectivity strength. Pearson’s correlation analysis with FDR (false discovery rate) correction was used to explore the relationships between structural network properties and FOG severity in PD FOG+ patients.

## Results

### Demographic and Clinical Characteristics

The demographic and clinical characteristics of all subjects are presented in Table 1. The analysis showed that no significant differences were found among PD FOG+ and PD FOG- patients and HCs concerning age, sex, education level, or Mini-Metal State Examination (MMSE) scores. In terms of Frontal Assessment Battery (FAB), The Timed Up and Go (TUG), and FOG questionnaire (FOGQ) scores, PD FOG+ patients exhibited significant differences in comparison to PD FOG- patients (*p* < 0.05). For UPDRS-III, H&Y scale, MoCA, HDRS, or HARS scores, there were no differences between PD FOG+ and PD FOG- patients.

**TABLE 1:** Demographic and clinical characteristics of participants.

Parameter	HC (*n* = 23)	PD FOG+ (*n* = 21)	PD FOG- (*n* = 34)	*P*-value
Age, years	64.2 ± 3.5	65.5 ± 6.3	64.7 ± 8.3	0.302^a^
Education, years	10.32 ± 2.4	9.46 ± 3.5	10.38 ± 4.3	0.39^a^
Sex, female/male	14/9	11/10	17/17	0.713^b^
Disease duration, years	NA	5.94 ± 5.35	3.13 ± 3.25	0.0063^c^
UPDRS-III	NA	23.42 ± 6.53	22.31 ± 9.36	0.21^c^
H&Y scale	NA	2.43 ± 0.53	2.13 ± 0.45	0.18^c^
FOGQ	NA	9.39 ± 5.62	1.24 ± 1.37	<0.001^c^*
MMSE	26.32 ± 1.53	25.55 ± 4.21	25.75 ± 4.35	0.576^a^
MoCA	NA	21.21 ± 4.43	21.53 ± 5.52	0.363^c^
FAB	NA	13.6 ± 2.4	15.9 ± 1.3	<0.001^c^*
TUG	NA	12.6 ± 1.4	1.7 ± 0.7	<0.001^c^*
HDRS	NA	7.84 ± 6.25	9.65 ± 6.24	0.35^c^
HARS	NA	11.43 ± 6.52	10.36 ± 7.45	0.76^c^

Data are shown as the mean ± standard deviation. *P < 0.05; ^a^one-way analysis of variance; ^b^chi-squared test; ^c^mean white U-test. FAB, Frontal Assessment Battery; FOGQ, Freezing of Gait Questionnaire; H&Y, Hoehn and Yahr; HARS, Hamilton Anxiety Rating Scale; HDRS, Hamilton Depression Rating Scale; HC, healthy control; MMSE, Mini-mental State Examination; MoCA, Montreal Cognitive Assessment; NA, not applicable; PD FOG+, Parkinson’s disease with freezing of gait; PD FOG-, Parkinson’s disease without freezing of gait; TUG, Timed Up and Go; UPDRS, Unified Parkinson’s Disease Rating Scale.

### The Small-World Properties of Structural Network

**FIGURE 3:**
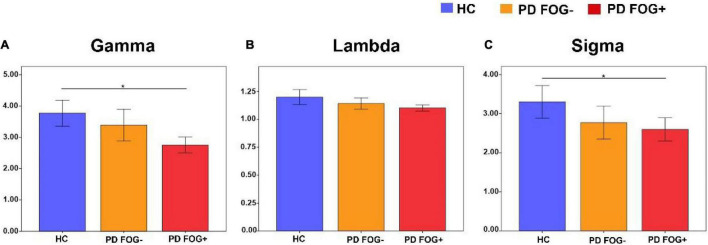
Small-world property comparison among the PD FOG+, PD FOG- and HC groups. **(A)** The normalized clustering coefficient (Gamma). **(B)** The normalized characteristic path length (Lambda). **(C)** The small-worldness (Sigma) (HC, healthy control; PD FOG- Parkinson’s disease without freezing of gait; PD FOG+, Parkinson’s disease with freezing of gait).

As shown in Figure 3, all three groups (PD FOG+, PD FOG-, HC) exhibited typical small-worldness topological properties (γ > > 1, λ≈1, δ > 1). Statistical comparison was used to detect whether there were significant differences in the small-world properties of the whole brain structural network among the three groups. Compared with HCs, PD FOG+ patients showed a significantly lower normalized clustering coefficient (γ) and small-worldness (δ) (*p* < 0.05). No significant differences were found in small-world (γ, λ, δ) properties between PD FOG- and HC or between PD FOG+ and PD FOG- (*P* > 0.05).

### The Global Properties of the Structural Brain Network

**FIGURE 4:**
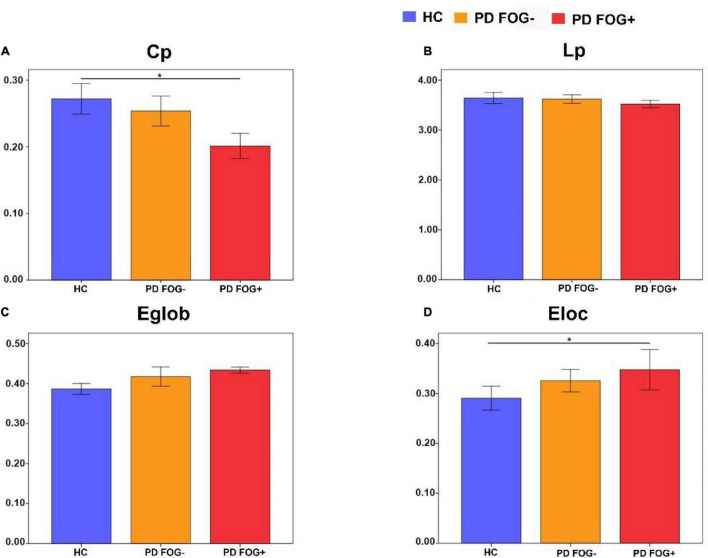
Comparisons of the global properties among the PD FOG+, PD FOG– and HC groups. **(A)** Clustering coefficient (C_p_). **(B)** Characteristic path length (L_p_). **(C)** Global efficiency (E_glob_). **(D)** Local efficiency (E_loc_).

Figure 4 shows that in terms of global properties, no significant differences were observed in L_p_ and E_glob_ among the three groups, but differences were found in C_p_ and E_loc_ between PD FOG+ and HC. PD FOG+ patients displayed significantly decreased C_p_ and increased E_loc_ compared with HCs. There was no significant difference in C_p_, L_p_, E_glob_, or E_loc_ between PD FOG- and HC or between PD FOG+ and PD FOG-.

### The Regional Properties of the Brain Network

**FIGURE 5:**
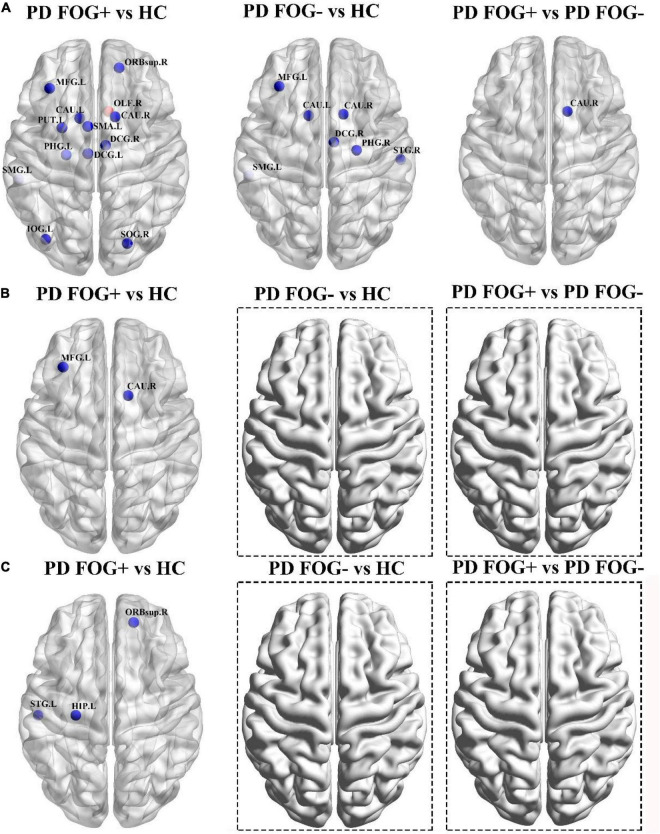
Brain regions with abnormal or significantly different properties among the PD FOG+, PD FOG– and HC groups. **(A)** Significantly different between centrality. **(B)** With significantly different degree centrality. **(C)** With significantly different nodal efficiency. The only red sphere indicates the increased centrality in PD FOG+ patients, and the other blue spheres indicate the decreased measures in the first group in the comparison. No brain regions with significant differences were observed for degree centrality and nodal efficiency in PD FOG– vs. HC and PD FOG+ vs. PD FOG–.

As shown in Figure 5A, significant differences in BC were found among the PD FOG+, PD FOG- and HC groups. PD FOG+ patients showed decreased BC in 12 brain regions including right superior frontal gyrus, left middle frontal gyrus, left supplementary motor area, left and right median cingulate, left parahippocampal gyrus, right superior occipital gyrus, left inferior occipital gyrus, left supramarginal gyrus, left and right caudate nucleus, left lenticular nucleus, increased BC in right olfactory cortex in PD FOG+ patients compared with HC (*p* = 0.029, *p* = 0.0032, *p* = 0.0047, *p* = 0.012, *p* = 0.039, *p* = 0.0008, *p* = 0.0064, *p* = 0.0058, *p* = 0.0027, *p* = 0.016, *p* = 0.0073, *p* = 0.0003, and *p* = 0.0053, respectively, FDR corrected). Compared with HCs, PD FOG- patients exhibited decreased BC in seven regions, including the left middle frontal gyrus, right median cingulate gyrus, right parahippocampal gyrus, left supramarginal gyrus, left and right caudate nucleus, and right superior temporal gyrus (*p* = 0.0039, *p* = 0.025, *p* = 0.0004, *p* = 0.0083, *p* = 0.0051, *p* = 0.031, *p* = 0.0016). Compared with PD FOG- patients, PD FOG+ patients showed decreased BC only in the left caudate nucleus (*p* = 0.0023).

For DC, PD FOG+ showed decreased values in the left middle frontal gyrus and right caudate nucleus (*p* = 0.036, *p* = 0.0065, Figure 5B). In terms of NE, PD FOG+ patients exhibited decreased values in the right superior frontal gyrus, left hippocampus, and left superior temporal gyrus (*p* = 0.0029, *p* = 0.017, *p* = 0.0010, Figure 5C). No differences were found in DC and NE between PD FOG+ and PD FOG- patients (*p* > 0.05).

### The White Matter Connectivity Between Groups

**FIGURE 6:**
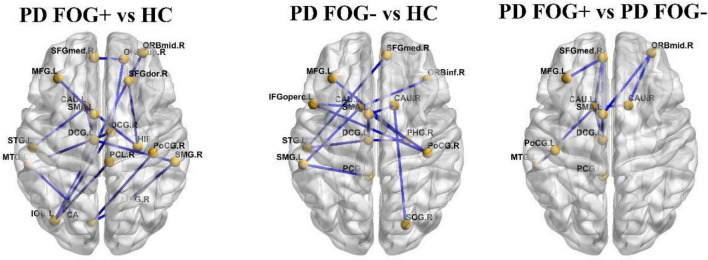
The connections with significantly different structural connectivity strengths among the PD FOG+, PD FOG–, and HC groups.

In Figure 6, based on the NBS method, significantly decreased structural connections were observed among the PD FOG+ vs. HC, PD FOG- vs. HC, and PD FOG+ vs. PD FOG- groups (FDR corrected). The subnetwork with 18 nodes and 18 edges showed decreased structural connections in PD FOG+ patients compared with HCs (left part of Figure 6). The related nodes included the right superior frontal gyrus (dorsolateral), right superior frontal gyrus (orbital part), left middle frontal gyrus, right middle frontal gyrus (orbital part), left supplementary motor area, left superior frontal gyrus (medial), left and right median cingulate, and paracingulate gyri, right hippocampus, left calcarine fissure and surrounding cortex, right lingual gyrus, left inferior occipital gyrus, right postcentral gyrus, right supramarginal gyrus, right paracentral lobule, left caudate nucleus, left superior temporal gyrus, and left middle temporal gyrus.

The PD FOG- patients showed decreased structural connections compared to HCs, and the subnetwork consisted of 13 nodes and 13 edges (the middle part of Figure 6). The nodes included the left middle frontal gyrus, left inferior frontal gyrus (opercular part), right inferior frontal gyrus (orbital part), left supplementary motor area, right superior frontal gyrus (medial), left median cingulate and paracingulate gyri, left posterior cingulate gyrus, right parahippocampal gyrus, right superior occipital gyrus, right postcentral gyrus, left supramarginal gyrus, right and left caudate nucleus, and left superior temporal gyrus.

Compared with PD FOG- patients, decreased structural connections were observed in PD FOG+ patients (the right part of Figure 6). The subnetwork with 10 nodes and 10 edges consists of the left middle frontal gyrus, right middle frontal gyrus (orbital part), left supplementary motor area, right superior frontal gyrus (medial), left median cingulate and paracingulate gyri, left posterior cingulate gyrus, left postcentral gyrus, left and right caudate nucleus, and left middle temporal gyrus.

### Relationships Between Network Properties and Freezing of Gait Severity

**FIGURE 7:**
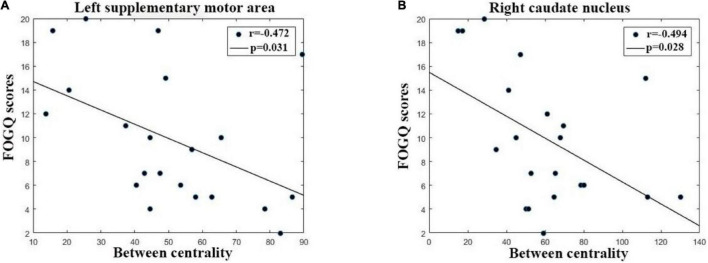
The correlation between FOGQ (freezing of gait questionnaire scores) and network topological properties in PD FOG+ patients. **(A)** The between centrality of the left supplementary motor area is negatively related to FOGQ scores. **(B)** The centrality of the right caudate nucleus was negatively related to FOGQ scores.

The relationships between network properties and the severity of FOG in PD FOG+ patients are shown in Figure 7. Regarding regional properties, the centrality in the left supplemental motor area (SMA) and right caudate nucleus (CAU) was negatively correlated with the FOGQ (*r* = –0.472, *p* = 0.031; *r* = –0.494, *p* = 0.028). There were no significant correlations between FOGQ scores and any other global and regional network properties.

## Discussion

By using DTI and graph theory network analysis, the topological organization of the structural brain network was compared among PD FOG+, PD FOG-, and HC. Four main findings have been found. First, at the global level, PD FOG+ patients showed significantly decreased γ and C_p_ compared with HCs. However, regarding local network efficiency (E_loc_), PD FOG+ patients are higher than HCs. These findings may imply a randomization shift of the structural brain network in PD FOG+. Second, at the regional level, significantly different topological properties, including BC, DC, and NE, have been found among PD FOG+, PD FOG-, and HC patients in several brain regions. Most importantly, PD FOG+ patients showed decreased BC in the left caudate nucleus. No other regions in regional topological properties have been found between PD FOG+ and PD FOG-. Third, disrupted structural connections were found among the three groups. Significantly decreased structural connections were observed among the three groups. Fourth, the regional parameters in several brain regions are significantly correlated with the severity of FOG. These findings may enable us to better understand the pathophysiological mechanism of FOG from the perspective of structural brain networks. In summary, the current study, to the best of our knowledge, is the first to systematically investigate the structural brain network among PD FOG+, PD FOG-, and HC.

### Different Global Topological Properties in Structural Networks

In this study, all PD FOG+, PD FOG-, and HC patients exhibited small-world organization, which is by previous functional network studies (Maidan et al., 2019; Ruan et al., 2020). The small-world properties were characterized by high local clustering and short path length, which reflect the best balance between local specialization and global integration (Achard et al., 2006; Bassett and Bullmore, 2017). In terms of global topological properties, PD FOG+ patients showed decreased C_p_ and increased E_loc_ compared with HCs. C_p_ is usually used to assess the information processing efficiency of brain networks, and the finding of decreased C_p_ in PD FOG+ patients may suggest a lower information processing ability. It is worth noting that a recent study using rs-fMRI also reported similar results (Li et al., 2021). Local efficiency is used to measure the ability of information transmission, and the increased E_loc_ may indicate a relatively higher ability of information transmission in PD FOG+ patients than in HCs. Disrupted global properties may reflect the miscommunication of overall information in PD FOG+ patients. In addition, this is the first time that graph theory has been used to explore the structural brain network of PD FOG+ patients.

### The Difference in Nodal Parameters Between Groups

In addition to global topological properties, significantly different regional parameters, including BC, DC, and NE, were found among the three groups. These regional parameters reflect the central role of nodes in the overall information communication of the brain network (Sporns et al., 2007). The changes in nodal topological properties reflect the abnormalities in regional neural circuits of the structural brain network, which can supply extra information that cannot be obtained from an investigation of the global topology of the network. These nodal properties have been widely used to characterize a variety of brain diseases, such as stroke and schizophrenia (Zhu et al., 2016; Cao et al., 2021).

Compared with HCs, decreased BC has been found in several brain regions, mainly located in the prefrontal cortex, supplementary motor area, hippocampus, cingulate gyrus, and occipital gyrus bilateral striatum, and increased centrality in the right olfactory cortex in PD FOG+ patients. Abnormalities in these brain regions have been reported by previous studies (Mitchell et al., 2019; Brugger et al., 2020; Ruan et al., 2020; Jin et al., 2021a). The prefrontal cortex plays an important role in cognitive and executive functions (Teffer and Semendeferi, 2012). The supplementary motor area is mainly involved in the movement produced and controlled by the body itself rather than the movement produced by external stimuli (Nachev et al., 2008). The hippocampus, cingulate gyrus, and occipital gyrus bilateral striatum are tightly related to spatial positioning, memory, and motor coordination functions (Kegeles et al., 2010; Knierim, 2015; Powell et al., 2018). Damage to their structural brain network may be one of the potential pathogeneses of FOG.

Moreover, significantly positive correlations were found between BC in the left SMA and right CAU and FOGQ scores, indicating that the symptoms of FOG will become more serious with the increase in BC in these two regions. The BC values of these two regions may be used as potential biomarkers. PD FOG- patients showed decreased BC in prefrontal and marginal lobes, the main difference between PD FOG+ patients and PD FOG- patients is that the BC decease of PD FOG- patients is not as serious as that of PD FOG+ patients.

PD FOG+ patients showed decreased DC and NE in the CAU, prefrontal cortex, hippocampus, and superior temporal gyrus (STG). STG is mainly responsible for processing auditory signals. Some studies have demonstrated the disrupted functional network properties of the STG in PD FOG+ patients (Ruan et al., 2020; Jin et al., 2021a). Compared with PD FOG- patients, PD FOG+ patients showed decreased BC in the right CAU. A resting-state functional MRI (rs-fMRI) study suggested that altered functional connectivity in the CAU may cause damage to the executive function of PD FOG+ patients (Steidel et al., 2021). Thus, the decreased BC in the right CAU may be an important aspect leading to FOG, which is also an important distinction between PD FOG+ and PD FOG-.

In our previous study on the functional brain network of FOG in PD, we found that PD FOG+ patients showed decreased DC in MFG, inferior temporal gyrus (ITG), STG, middle temporal gyrus (MTG), and parahippocampal gyrus (PhG) (Jin et al., 2021a). These findings suggest that PD FOG+ patients have some commonalities and differences in brain regions with impaired functional and structural brain networks. The commonalities are represented by the disrupted MFG, STG and PhG. This may indicate that the structure and function of the brain are closely related and abnormal brain structure is accompanied by the weakening of the corresponding function. In another work (Jin et al., 2021b), we found significant correlations between structural and functional parameters by employing tract-based spatial statistical analysis and voxel-mirrored homotopic connectivity methods. The differences are reflected by the abnormal CAU, SMG, BCG, and SOG, ORBsup regions (observed in structural network, but not in functional network) and the disrupted ITG (observed in functional network, but not in structural network). It indicates that different definitions of network connections have a wide range of influences on network topology (Liang et al., 2009; Korhonen et al., 2021). Therefore, how determining a reasonable connectivity measure for the nature of brain connectivity becomes an important issue in this field.

In conclusion, a comprehensive quantitative assessment of the similarity and specificity of structural and functional brain networks combined with multimodal imaging techniques remains an important direction for current research (Park and Friston, 2013; van Diessen et al., 2013).

### Distinctive Connectivity Characteristics Between Groups

Significantly decreased structural connections were found in the PD FOG+ and PD FOG- groups. These brain regions were mainly located in the frontal lobe, occipital lobe, temporal lobe, parietal lobe, and marginal lobe. It is well known that the early symptoms of Parkinson’s disease are a decrease in dopamine input into the cortex and subcortical structures (Rodriguez-Oroz et al., 2009). The decreased structural connections in PD FOG+ patients are consistent with previous tract-based spatial statistic (TBSS) findings (Iseki et al., 2015; Wang et al., 2016). Decreased FA values have been found in widespread cortical and subcortical brain regions, including the frontal lobe, hippocampus, striatum, internal capsule, and cerebellum (Pietracupa et al., 2018; Bharti et al., 2019). Using the NBS method, functional network-based rs-fMRI has found decreased functional connections in the sensorimotor cortex, visual network, default mode network, auditory network, dorsal attention network, subcortical regions, and limbic network (Ruan et al., 2020).

More importantly, we found that PD FOG+ patients showed significantly decreased structural connections in the frontal pole, temporal pole, parietal lobe, and marginal lobe compared with PD FOG- patients. Those findings may indicate that PD FOG+ patients showed more disrupted structural connections. Based on structural connectivity and topological properties of brain network among the three groups, we found that PD FOG+ patients displayed disrupted structural connectivity and topological properties compared with PD FOG- and HC groups. Especially, PD FOG+ patients showed decreased BC in CAU, which is related to the severity of FOG, and decreased structural connectivity. These findings may indicate that during the transition from PD FOG- to PD FOG+, there may be changes in structural connectivity (edge) and topological properties of the brain region (node), and these altered regions should receive more clinical attention. Both nodes and edges are fundamental components of brain networks, and node-centric and edge-centric network models are regarded as complementary approaches to revealing the organizational characteristics of the nervous system (Stanley et al., 2013; Faskowitz et al., 2020, 2022). In addition, we also found no significant differences in global parameters between PD FOG+ and PD FOG-, possibly because regional brain abnormalities did not extend to global changes. These results may provide a potential basis for formulating clinically targeted treatments.

### Limitations

There are several limitations to our study. First, the sample size of all subjects was relatively small, which may reduce the statistical ability. Second, our study is cross-sectional; therefore, the dynamic alterations of related regions and measures cannot be examined during the progression of PD FOG+ from PD FOG-. Third, this is the first study to use graph theory to explore the topological properties of the structural brain network in PD FOG+ patients, and the results need to be further tested to prove their validity.

## Conclusion

**FIGURE 8:**
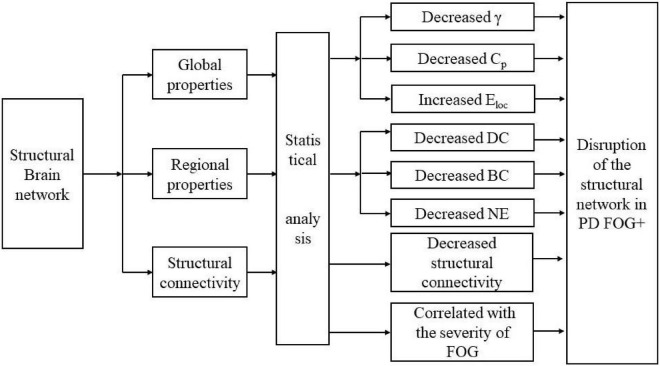
Schematic diagram of the main conclusions.

The structural brain network of the PD FOG+, PD FOG-, and HC groups showed small worldness properties. As displayed in Figure 8, compared with HCs, PD FOG+ patients showed decreased structural connections, γ, and CE, and increased N_loc_. PD FOG+ patients exhibited decreased BC, DC, and NE in several regions, and these brain regions were mainly located in the prefrontal cortex, supplementary motor area, hippocampus, cingulate gyrus, and occipital gyrus bilateral striatum. Compared with PD FOG-, PD FOG+ patients showed significantly decreased BC in the right CAU and decreased structural connections in the frontal pole, temporal pole, parietal lobe, and marginal lobe. These results may suggest the disruption of the structural network in PD FOG+. These findings may help understand the pathophysiological mechanism of FOG and provide new ideas for studying the neurobiology of FOG. The topological properties of abnormal brain regions can be used as potential biomarkers, which will help the early diagnosis, identification, and treatment of PD FOG+ patients.

## Data Availability Statement

The datasets presented in this article are not readily available because of the requirements of the Ethics Committee of Guangzhou First People’s Hospital. Requests to access the datasets should be directed to XW.

## Ethics Statement

The studies involving human participants were reviewed and approved by the Ethics Committee of Guangzhou First People’s Hospital. The patients/participants provided their written informed consent to participate in this study.

## Author Contributions

CJ performed the experiments and analyzed the data along with SQ and LY. SQ, YT, YY, and XW conceived the study, presented the results, and wrote the manuscript along with CJ. XR collected and analyzed the data. YT and CL supervised the algorithm development and analyzed the data. All authors read and approved the final manuscript.

## Conflict of Interest

The authors declare that the research was conducted in the absence of any commercial or financial relationships that could be construed as a potential conflict of interest.

## Publisher’s Note

All claims expressed in this article are solely those of the authors and do not necessarily represent those of their affiliated organizations, or those of the publisher, the editors and the reviewers. Any product that may be evaluated in this article, or claim that may be made by its manufacturer, is not guaranteed or endorsed by the publisher.

## Footnotes

1
https://www.ibm.com/products/spss-statistics
